# Overall survival and associated factors in women with metastatic breast cancer treated with trastuzumab at a public referral institution

**DOI:** 10.1590/1980-549720230045

**Published:** 2023-10-20

**Authors:** Débora Silva Gonçalves, Arn Migowski, Susanne Crocamo Ventilari da Costa, Rodrigo Saar da Costa, Katia Marie Simões e Senna, Ivan Ricardo Zimmermann

**Affiliations:** I Instituto Nacional de Cardiologia, Professional Master's Program in Health Technology Assessment – Rio de Janeiro (RJ), Brazil.; II Instituto Nacional do Câncer José Alencar Gomes da Silva – Rio de Janeiro (RJ), Brazil.; III Universidade de Brasília, School of Health Sciences, Department of Collective Health – Brasília (DF), Brazil.

**Keywords:** Breast neoplasms, Neoplasm metastasis, Survival analysis, Trastuzumab, Unified health system, Neoplasias da mama, Metástase neoplásica, Análise de sobrevida, Trastuzumab, Sistema único de saúde

## Abstract

**Objective:**

To characterize associated factors and overall survival of women with metastatic breast cancer treated with trastuzumab after its incorporation into the SUS, and additionally to present the direct costs of this technology.

**Methods:**

This is a retrospective cohort, based on data from computerized medical records from one of the units of the National Cancer Institute (INCA), in Rio de Janeiro-RJ, Brazil. Women with HER-2 positive metastatic breast cancer undergoing trastuzumab treatment from September 2017 to August 2018 were included. Overall survival was estimated using the Kaplan-Meier method and compared between groups using the log-rank test.

**Results:**

136 women were selected, whose median age at diagnosis was 51 years (range: 21–81 years). The median OS was 43.63 months (95%CI 33.92–53.34). It is observed that the median OS for the population already diagnosed with metastatic disease (stage IV) was significantly lower than for patients diagnosed in stages I-III (37.43 months vs. 48.6 months, p<0, 01). Women without previous use of trastuzumab had a higher median OS than patients pretreated with trastuzumab (45.16 months vs. 40.73 months, p<0.01).

**Conclusion:**

Trastuzumab improves survival in HER-2 positive metastatic breast cancer. Brain and multiple metastases are associated with a worse prognosis. It is essential to avoid advanced staging and perform surgical treatment, with emphasis on radical mastectomy. The SUS must adopt policies and strategies for early diagnosis and guarantee access to trastuzumab, considering its high cost.

## RESUMO

**Objetivo:**

Caracterizar fatores associados e sobrevida global de mulheres com câncer de mama metastático tratadas com trastuzumabe, após sua incorporação ao Sistema Único de Saúde, e apresentar os custos diretos dessa tecnologia.

**Métodos:**

Trata-se de uma coorte retrospectiva, baseada em dados de prontuários do Instituto Nacional do Câncer, no Rio de Janeiro (RJ). Foram incluídas mulheres com câncer de mama metastático HER-2 positivo em tratamento com trastuzumabe no período de setembro de 2017 a agosto de 2018. A sobrevida global foi estimada pelo método Kaplan-Meier e comparada entre grupos pelo teste de *log-rank*.

**Resultados:**

Foram selecionadas 136 mulheres, cuja mediana da idade do diagnóstico foi de 51 anos (amplitude: 21-81 anos). A mediana da sobrevida global foi de 43,63 meses (IC95% 33,92–53,34). Observa-se que a mediana da sobrevida global para a população já diagnosticada com doença metastática (estádio IV) foi significativamente menor do que para as pacientes diagnosticadas nos estádios I-III (37,43 meses vs. 48,6 meses, p<0,01). Já mulheres sem uso prévio de trastuzumabe apresentaram uma mediana de sobrevida global maior do que as pacientes pré-tratadas com trastuzumabe (45,16 meses vs. 40,73 meses, p<0,01).

**Conclusão:**

O trastuzumabe aumentou a sobrevida no câncer de mama metastático HER-2 positivo. Metástases cerebrais e múltiplas mostraram-se associadas a um pior prognóstico. É fundamental evitar o estadiamento avançado e realizar tratamento cirúrgico, destacando-se a mastectomia radical. O Sistema Único de Saúde deve adotar políticas e estratégias para o diagnóstico precoce e garantir acesso ao trastuzumabe, considerando seu alto custo.

## INTRODUCTION

Cancer is a public health problem whose incidence and mortality is increasing worldwide^1^. Several factors contribute to the increase in its economic burden, with the cost of antineoplastic drugs being a high-impact component^2^. Consistent with a worldwide trend, breast cancer is the most common among women in all Brazilian regions (excluding non-melanoma skin cancer)^1^.

In the metastatic phase, breast cancer affects other organs in addition to the primary site and is indicated for palliative treatment, whose main therapeutic objective is to increase overall survival (OS) and progression-free survival (PFS) and improve quality of life related to health^3–5^. Among the agents available in the therapeutic arsenal for breast cancer, trastuzumab stands out, a biological antineoplastic drug that blocks the type 2 receptor of the human epidermal growth factor (HER-2)^6^.

International studies indicate that the addition of trastuzumab is associated with a positive impact on the OS of patients with breast cancer, including those with metastatic disease^7–11^. In Brazil, trastuzumab was incorporated into the Brazilian Unified Health System (SUS) in 2012 for the treatment of early and locally advanced breast cancer^12,13^. Later, in 2017, it was decided to expand the incorporation of trastuzumab for the treatment of metastatic breast cancer^14^.

The National Commission for the Incorporation of Technologies (*Comissão Nacional de Incorporação de Tecnologias* – CONITEC) in SUS points out that post-incorporation monitoring is an important step in the process of health technologies assessment (HTA), which aims to assess the effectiveness of a given technology through real life data^15^. Real-life studies use records from clinical practice, unlike randomized clinical trials (RCTs), which are conducted under ideal, controlled conditions that may limit diversity in the general population. Therefore, despite implying limitations in the origin and reliability of the data, there is a tendency to value these studies with real-world data as important sources of scientific evidence^16^.

In Brazil, there is a shortage of studies on survival in metastatic breast cancer, especially specific studies on the population using trastuzumab. Therefore, the innovative nature and relevance of this work is highlighted, as it focuses specifically on Brazilian women with metastatic disease treated with trastuzumab, thus contributing to the monitoring of this technology after its incorporation into SUS. The objective of the study was to characterize the associated factors and the OS of women with HER-2 positive metastatic breast cancer treated with trastuzumab, after its incorporation into SUS, and to estimate the direct costs of acquiring this technology.

## METHODS

### Study context and design

This is a retrospective cohort study based on data from electronic medical records of a public hospital that is a reference in breast oncology in Rio de Janeiro (RJ), the National Cancer Institute (*Instituto Nacional de Câncer* – INCA).

### Participants

The study population consisted of female patients with metastatic breast cancer and HER-2 overexpression who were treated with trastuzumab alone or in combination between September 2017 and August 2018, the period corresponding to the first year following the incorporation of trastuzumab for metastatic breast cancer by CONITEC.

### Variables and data sources

After obtaining permission to access the institution's computerized medical records, data collection was based on a standardized electronic form, which followed the recommendations of the Strengthening the Reporting of Observational Studies in Epidemiology (STROBE)^17^ guide for reporting observational studies. The variables were: date of diagnosis of the first metastasis, date of death or last visit to the institution, age at initial diagnosis, staging at initial diagnosis, skin color, hormone receptor, histopathological grade, metastatic site, type of surgery, body mass index (BMI), prior exposure (adjuvant and/or neoadjuvant) to trastuzumab, and dose of each cycle of trastuzumab. Patients with incomplete data were excluded from the sample. Weight and height were collected at two moments (beginning and end of treatment), and then the average of these two values was calculated as the basis for calculating the BMI. The assessment of the occurrence of death was verified in the medical records and confirmed by consulting the official records of births and deaths of the judiciary power of the state of Rio de Janeiro^18^. OS consisted of the time between the diagnosis of the first metastasis until the occurrence of the outcome death from any cause or the last attendance at the institution, considering the deadline of December 31^st^, 2021 (censored).

To account for the cost of treatment with trastuzumab, the calculation was restricted to the time frame established for the study (12 months). It should be noted that the institution recommends that the start of trastuzumab be associated with chemotherapy, and, in the maintenance phase, it is possible to associate it with hormone therapy or to carry it out as a monotherapy, so that the same patient can go through these three therapeutic modalities, and, therefore, the effect of combining trastuzumab with chemotherapy or hormone therapy has not been evaluated. The drug price was obtained by the Federal Government's Health Price Bank (*Banco de Preços em Saúde* – BPS) considering the weighted average of the prices charged on the basis of the Integrated System of General Services Administration (*Sistema Integrado de Administração de Serviços Gerais* – SIASG)^19^. For this purpose, price records available in the period from 2012 to 2021 in purchases by the Department of Healthcare Logistics (*Departamento de Logística em Saúde* – DLOG) of the Ministry of Health (MoH) for the presentation of 150 mg and by federal institutions of the state of Rio de Janeiro for the presentation of 440 mg were considered.

### Statistical methods

OS was estimated using the Kaplan-Meier method and compared between groups using the log-rank test. Seeking to eliminate the confounding bias, multiple regression models were also constructed using Cox proportional regression to estimate the risk ratios (hazard) and the 95% confidence intervals of the magnitude of association of the possible associated factors. The presence of multicollinearity between the variables was verified using a Spearman correlation matrix (Supplementary Material – S1). The exclusion criterion for multicollinearity was the value of r≤0.50 (strong correlation) when p≤0.05, prevailing the variable with greater statistical significance. Inclusion criteria for the multiple model was p≤0.10 for the log-rank test and for the hazard ratio (HR) of the univariate analysis. The statistical significance resulting from the multivariate analysis considered p≤0.05. Cox-Shell residual analysis was conducted to assess the adequacy of the adjusted model (Supplementary Material – S2). The analyses were performed using the R language with the aid of the free interface Jamovi 2.2.5 and the SPSS software.

### Ethical aspects

The ethical aspects involved are in accordance with Resolution No. 466, of December 12^th^, 2012, and its complements, and the study was approved by the local research ethics committee (Protocol No. 27316619.4.0000.5274).

## RESULTS

### Baseline characteristics

Records of 149 women with metastatic breast cancer undergoing treatment with trastuzumab during the analyzed period were identified. However, 13 patients (8.72%) were excluded due to records with incomplete data. Therefore, the final study population consisted of 136 patients. Table 1 presents the epidemiological profile, providing a summary of the clinical and pathological characteristics of the population.

**Table 1:** Epidemiological characterization of the population with metastatic breast cancer using trastuzumab from 09/2017 to 08/2018 in a reference hospital in oncology in Rio de Janeiro, Brazil.

Explanatory variables		Staging at diagnosis		
		(I–III) n (%)	IV n (%)	Total n (%)
Age at diagnosis				
	20–29	2 (2.7)	3 (4.8)	5 (3.7)
	30–49	37 (50.8)	25 (39.7)	62 (45.6)
	50–69	32 (43.8)	30 (47.6)	62 (45.6)
	70–90	2 (2.7)	5 (7.9)	7 (5.1)
Skin color				
	White	30 (41.1)	21 (33.3)	51 (37.5)
	Black and brown	43 (58.9)	42 (66.7)	85 (62.5)
Metastatic site				
	1_bone	11 (15.1)	10 (15.8)	21 (15.4)
	2_CNS	7 (9.6)	2 (3.2)	9 (6.6)
	3_Liver	3 (4.1)	3 (4.8)	6 (4.4)
	4_Multiple	34 (46.6)	36 (57.1)	70 (51.6)
	5_Lung	6 (8.2)	3 (4.8)	9 (6.6)
	6_Others	12 (16.4)	9 (14.3)	21 (15.4)
Hormone receptor (estrogen or progesterone)				
	Negative	28 (38.4)	20 (31.5)	48 (35.3)
	Positive	45 (61.6)	43 (68.2)	88 (64.7)
Prior exposure to trastuzumab				
	Exposed	41 (56.2)	7 (11.2)	48 (35.3)
	Not exposed	32 (43.8)	56 (88.8)	88 (64.7)
Types of surgery				
	1_None	13 (17.8)	36 (57.1)	49 (36.0)
	2_Radical mastectomy	38 (52.0)	03 (4.8)	41 (30.1)
	3_Simple mastectomy	03 (4.1)	02 (3.2)	05 (3.7)
	4_Breast conservative	07 (9.7)	-	07 (5.2)
	5_Only secondary tumors	03 (4.1)	10 (15.9)	13 (9.6)
	6_Others only (vascular access support)	09 (12.3)	12(19.0)	21 (15.4)
Histopathological degree				
	G1	01 (1.3)	05 (7.9)	06 (4.4)
	G2	38 (52.1)	36 (57.2)	74 (54.4)
	G3	34 (46.6)	22 (34.9)	56 (41.2)
Body mass index				
	Normal weight (18.5 to 24.9)	20 (27.4)	18 (28.6)	38 (27.9)
	Overweight (25.0 to 29.9)	31 (42.5)	25 (39.7)	56 (41.2)
	Obesity (≥30.0)	22 (30.1)	20 (31.7)	42 (30.9)

The median age at the time of primary diagnosis was 51 years, ranging from 21 to 81 years. Mean body weight was 70.75 kg (standard deviation: 13.11), mean height was 158 cm (standard deviation: 0.06), and mean BMI was 28.3, indicating overweight. Most patients (62.5%) self-declared as brown or black. Regarding staging, it was observed that 46.3% of the cases were already diagnosed in stage IV, indicating metastases. Among patients with single distant metastases, the most common sites were bone (15.4%), lungs (6.6%), central nervous system (6.6%), and liver (4.4%). Multiple metastases were observed in 51.6% of the sample.

### Overall survival and associated factors

**Figure 1:**
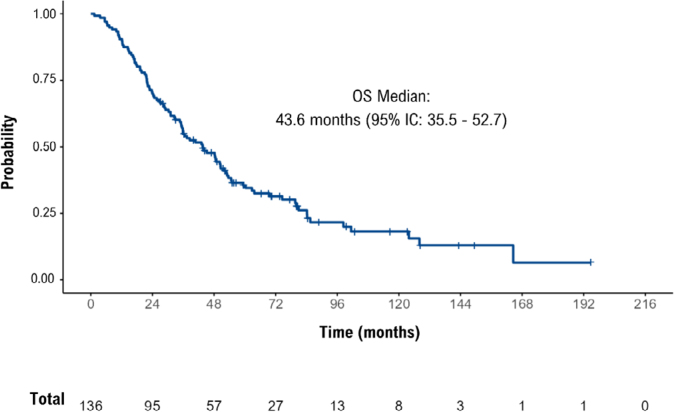
Probability of overall survival according to the Kaplan-Meier method for the general population of patients with metastatic breast cancer using trastuzumab from 09/2017 to 08/2018 in a reference hospital in oncology in Rio de Janeiro, Brazil.

**Figure 2:**
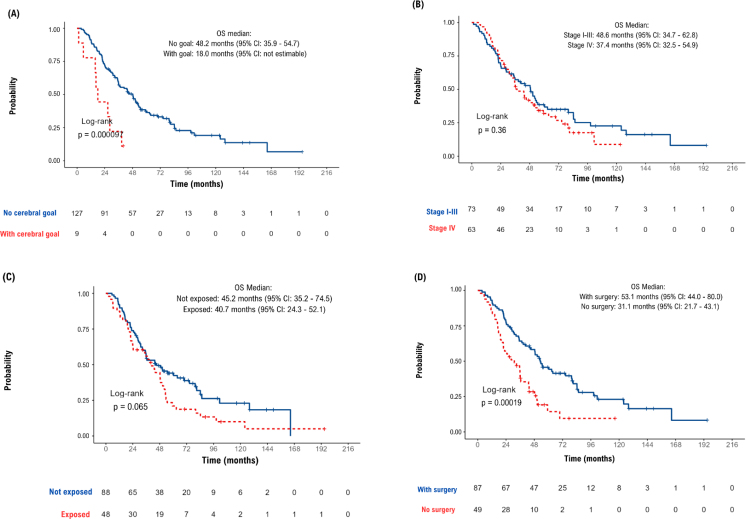
Subgroup analysis for the probability of overall survival according to the Kaplan-Meier method for patients with metastatic breast cancer using trastuzumab from 09/2017 to 08/2018 at a reference hospital in oncology in Rio de Janeiro, Brazil.

The probability of patients with metastasis at diagnosis surviving up to five years was 34.9%, and for those who presented metastasis during the course of the disease, it was 41.1%. Approximately 6% of patients survived 10 years or more. The median OS for the general study population was 43.6 months (95%CI 35.5–52.7) (Figure 1). In a subgroup analysis (Figure 2), the median OS for patients diagnosed with metastatic disease (stage IV) was 37.4 months (95%CI 32.5–54.9), while for those diagnosed with stages I -III, median OS was 48.6 months (95%CI 34.7–62.8). Patients without prior exposure to trastuzumab had a median OS of 45.2 months (95%CI 35.2–74.5), while those previously exposed had a median of 40.7 months (95%CI 24.3–52.1). In cases of brain metastasis, the median OS was 18 months (95%CI: not estimable), whereas, when there was no brain metastasis, the median OS was 47.2 months (95%CI 35.9–54.7). When it was observed that no surgical procedure was performed, the median OS was 31.1 months (95%CI 21.7–43.1); in cases where a surgical procedure was performed, the median OS was 53.1 months (95%CI 44.0–80.0).

Furthermore, Table 2 reveals the results of the univariate analysis, demonstrating that having brain metastases (HR: 4.86; 95%CI 1.99–11.89; p=0.001) or multiple metastases (HR: 1.92; 95%CI % 1.07–3.45; p=0.029) is significantly associated with lower OS. The absence of prior exposure to trastuzumab in adjuvant and/or neoadjuvant therapy showed a trend of association with longer survival (HR: 0.69; 95%CI 0.46–1.03; p=0.067). Patients undergoing radical mastectomy (HR: 0.38; 95%CI 0.23–0.64, p<0.0001), surgery for secondary tumors (HR: 0.36; 95%CI 0.17–0.78, p=0.010), and surgeries to support vascular access (HR: 0.54; 95%CI 0.30–0.98, p=0.044) had higher survival rates. Although the variable age at diagnosis showed a tendency for very young patients (≤29 years) diagnosed with metastatic breast cancer to have a worse prognosis, there was not enough statistical power to make this statement.

**Table 2:** Univariate analysis of overall survival in metastatic breast cancer using trastuzumab from 09/2017 to 08/2018 in a reference hospital in oncology in Rio de Janeiro, Brazil.

Explanatory variables		HR (univariate)	log-rank
Age at diagnosis			
	20–29	-	p=0.62
	30–49	0.64 (0.25–1.61. p=0.340)	
	50–69	0.80 (0.32–2.01. p=0.633)	
	70–90	0.83 (0.25–2.74. p=0.762)	
Skin color			
	White	-	p=0.15
	Black and brown	1.36 (0.90–2.06. p=0.147)	
Metastatic site			
	1_bone	-	p=0.00051
	2_CNS	4.86 (1.99–11.89. p=0.001)	
	3_Liver	2.42 (0.86–6.76. p=0.092)	
	4_Multiple	1.92 (1.07–3.45. p=0.029)	
	5_Lung	0.68 (0.22–2.07. p=0.496)	
	6_Others	0.99 (0.45–2.19. p=0.978)	
Hormone receptor (estrogen or progesterone)			
	Negative	-	p=0.26
	Positive	0.79(0.52–1.19. p=0.259)	
Prior exposure to trastuzumab			
	Exposed	-	p=0.065
	Not exposed	0.69 (0.46–1.03. p=0.067)	
Type of surgery			
	1_None	-	p=0.0041
	2_Radical mastectomy	0.38 (0.23–0.64. p<0.001)	
	3_Simple mastectomy	0.72 (0.28–1.85. p=0.491)	
	4_Conservative	0.77 (0.30–1.95. p=0.577)	
	5_Secondary tumors	0.36 (0.17–0.78. p=0.010)	
	6_Others (vascular access support)	0.54 (0.30–0.98. p=0.044)	
Histopathological degree			
	G1	-	p=0.39
	G2	2.08 (0.65–6.66. p=0.217)	
	G3	1.78 (0.55–5.77. p=0.337)	
Body mass index			
	Normal weight (18.5 to 24.9)	-	p=0.43
	Overweight (25.0 to 29.9)	0.74 (0.46–1.20. p=0.219)	
	Obesity (≥30.0)	0.92 (0.56–1.52. p=0.757)	
Staging			
	I–III	-	p=0.36
	IV	1.20 (0.81–1.80. p=0.365)	

A moderate correlation was observed between the variables of previous exposure to trastuzumab and staging (r=0.375; p<0.001), as well as a weak correlation between hormone receptor and metastatic site factors (r=0.190; p<0.027) (Supplementary Material – S1). However, no strong correlations were found between the variables, which ruled out the exclusion of any variable given the presence of multicollinearity.

**Figure 3:**
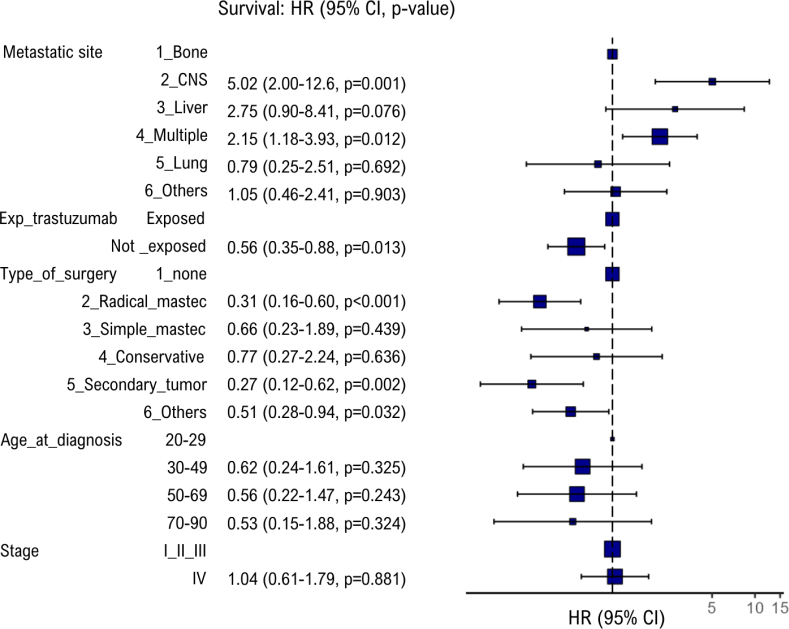
Forest plot of the multivariate analysis for overall survival of patients with metastatic breast cancer using trastuzumab from 09/2017 to 08/2018 in a reference hospital in oncology in Rio de Janeiro, Brazil (scenario 2).

The multivariate model was developed considering two scenarios: the first included only the variables that met the inclusion criteria established in the methodology (Supplementary Material – S3), while the second added the factors of age at initial diagnosis and staging given the biological and epidemiological plausibility, as shown in Figure 3. In both scenarios, the association of factors of previous exposure to trastuzumab, the metastatic site, and the type of surgery maintained their significance and magnitude.

### Economic analysis

A significant reduction in the acquisition price of trastuzumab is evident from 2020 for both presentations, 440 mg and 150 mg. In addition, over the 10 years analyzed, an important difference in price between these two presentations was observed, favoring the 150 mg vial (Supplementary Material – S4).

During the 12-month period, each study participant consumed, on average, 3,245.32 mg of trastuzumab, divided into an average of seven doses per patient. This resulted in an average dose of 463.61 mg. Considering the average price of 1 mg of trastuzumab in 2017 (R$20.97), a cost of R$9,721.90 is estimated per dose. However, with the average price of 1 mg of trastuzumab in 2021 (R$5.85), the cost of a dose of this drug would be R$2,712.11, representing a 72.1% reduction in this cost.

## DISCUSSION

### Main findings

This study examined the probability of survival and associated factors in patients with HER-2 positive metastatic breast cancer treated with trastuzumab at a public cancer institution in Brazil. The OS in this type of cancer varies according to late diagnosis and prognostic factors related to the aggressiveness of the disease^20,21^. However, the results found are consistent with the assumption of improved survival with trastuzumab treatment.

A French study^8^ analyzed patients with metastatic breast cancer and found a median OS of 44.91 months (95%CI 42.51–47.90) in the subgroup of 2,861 HER-2 positive patients. The study suggests that the improvement in OS can be attributed to the development of drugs that target HER-2, such as trastuzumab^8^. Our findings also corroborate these results, with a median OS of 43.63 months.

An Australian observational study^9^ demonstrated a median OS of 21.8 months for patients pretreated with trastuzumab and 35.6 months for patients without prior use, which is consistent with our findings of a lower median OS in pretreated patients compared to those not exposed to the drug (40.73 months *vs*. 45.16 months). However, it is important to highlight that the calculation of OS in the aforementioned study considered the date of initiation of trastuzumab use, while the present study considered the date of diagnosis of the first metastasis, which may have influenced the results obtained. An additional study^10^ found a median OS of 48.2 months in patients with metastatic breast cancer previously exposed to trastuzumab, which is in line with the results of this study. These results indicate that resistance to trastuzumab may be a contributing factor to lower survival in pretreated patients compared to those who did not receive prior treatment.

Two Brazilian studies reported that between 5.1 and 8.7% of patients with breast cancer were diagnosed at stage IV^9,22^. In a specific study on metastatic breast cancer, a percentage of 26.6% of diagnoses was found in stage IV, closer to the results of this study, in which 46.3% of patients were diagnosed in this stage^23^. These findings highlight the need for more research on metastatic breast cancer in the Brazilian population, especially in the context of SUS, for better understanding and early intervention.

Analysis of the results revealed a downward trend in OS as the staging progressed in terms of both the five-year survival rate (41.1% for stages I to III, and 34.9% for stage IV) and the median of survival (48.6 months for stages I to III, and 37.43 months for stage IV). These findings are in line with previous studies that highlight the influence of staging on breast cancer survival, including metastatic ones^23–25^. Furthermore, an American study^26^ reported a lower percentage of stage IV diagnoses (28%) compared to this study (46.3%), suggesting a possible later diagnosis in Brazil^27^. Furthermore, it was observed that approximately 6% of the analyzed population survived for 10 years or more, compared to 11% in the American study^26^.

The literature shows that the prognosis of metastatic breast cancer varies according to the metastatic site, with bone being the most common, followed by liver, lung, and brain^22,28,29^. The data collected in our sample confirmed this trend. Analyses performed indicated that patients with brain metastases had the worst prognosis in all scenarios, followed by multiple metastases. These results are in agreement with other studies that also show lower OS in women with brain and multiple metastases^20,21,28^.

Young patients (≤29 years old) diagnosed with metastatic breast cancer tended to have a worse prognosis, which is in line with previous Brazilian studies^30,31^. These studies showed that young women with breast cancer have unfavorable clinical and pathological characteristics, such as more aggressive subtypes, and tend to have more advanced stages at breast cancer diagnosis^30,31^. However, when age groups of very young patients (less than 40 years old) are excluded, the prognosis is expected to worsen with advancing age^31^.

The present work highlights the importance of adequate surgical treatment in breast cancer, which can be reiterated by another Brazilian study that pointed to the delay in performing surgery for curative purposes as a factor associated with increased mortality from the disease^22^. Furthermore, in agreement with our findings, a comparative study demonstrated that radical mastectomy, with the removal of at least ten lymph nodes, is associated with an increase in survival compared to the group without surgery [HR 0.63 (95%CI 0.50–0.80), p<0.001]^32^. This evidence reinforces the importance of early diagnosis and adequate surgical treatment in breast cancer.

Trastuzumab has an unequivocal benefit in increasing the OS of HER-2 positive metastatic breast cancer^10–13^. However, its high cost can make it difficult for the population to access this technology and affect health systems. Brazilian studies have shown that trastuzumab was the drug with the greatest financial impact for INCA^33^ and represented one of the highest expenses in the judicialization of antineoplastic drugs in a federal hospital in Rio Grande do Sul^34^. This highlights the issue of the high cost associated with trastuzumab.

This study corroborates previous research that demonstrated that the reduction in the price of trastuzumab did not occur immediately after partnerships for productive development were signed^35^. However, recent analyses of prices practiced in 2020 and 2021 showed a significant reduction for both presentations of trastuzumab compared to 2017 prices, evidencing the likely impact of the development of biosimilars^35,36^. Currently, there are six trastuzumab biosimilars registered and approved by the National Health Surveillance Agency (*Agência Nacional de Vigilância Sanitária* – ANVISA) in Brazil^37^. It is important to emphasize that biosimilars are important alternatives to expand access to high-cost treatments. However, there are still concerns in the medical community regarding the safety, immunogenicity, extrapolation, and interchangeability of treatment regimens related to these biosimilar drugs^38^.

### Strengths and limitations

This study has significant advantages, based on real-world evidence, looking at the technology's performance under uncontrolled conditions. This highlights the importance of post-incorporation monitoring studies to assess the impact of technologies adopted by SUS. The study focuses on patients with metastatic breast cancer, an area with scarce evidence in the Brazilian population, and was carried out in a breast cancer reference hospital, from the perspective of SUS.

This study has important limitations. Sample size may not have been sufficient to identify important associations in multiple regression models. Future studies with larger samples may confirm these findings. The diversity of participants in an uncontrolled environment and the specific approach from the SUS perspective require caution regarding external validity. Potential information bias due to the retrospective and observational nature of the study may interfere, even with statistical adjustments.

There was confirmation that trastuzumab increased survival in women with HER-2 positive metastatic breast cancer, especially without prior drug exposure. Metastatic site has a significant impact on survival, with brain and multiple metastases associated with a worse prognosis. Avoiding advanced staging and performing adequate surgical treatment, especially radical mastectomy, is also an important factor in increasing survival. Therefore, it is essential that SUS adopt public policies and strategies that promote early diagnosis and guarantee access to trastuzumab, considering its high cost.
