# ERRATUM

**DOI:** 10.1590/1980-549720230045erratum

**Published:** 2023-12-04

In the manuscript “Overall survival and associated factors in women with metastatic breast cancer treated with trastuzumab at a public referral institution”, DOI: https://doi.org/10.1590/1980-549720230045, published in the Rev Bras Epidemiol. 2023; 26: e230045:

**Where it reads:**

Arn Migowski Rocha dos Santos^I^

**It should read:**

Arn Migowski^I,II^

**Where it reads:**

HOW TO CITE THIS ARTICLE: Gonçalves DS, Santos AMR, Costa SCV, Costa RS, Senna KMS, Zimmermann IR. Overall survival and associated factors in women with metastatic breast cancer treated with trastuzumab at a public referral institution. Rev Bras Epidemiol. 2023; 26: e230045. https://doi.org/10.1590/1980-549720230045

**It should read:**

HOW TO CITE THIS ARTICLE: Gonçalves DS, Migowski A, Costa SCV, Costa RS, Senna KMS, Zimmermann IR. Overall survival and associated factors in women with metastatic breast cancer treated with trastuzumab at a public referral institution. Rev Bras Epidemiol. 2023; 26: e230045. https://doi.org/10.1590/1980-549720230045

**Where it reads:**

AUTHORS’ CONTRIBUTIONS: Gonçalves, D.S.: Conceptualization, Data curation, Writing - original draft, Methodology, Software. Santos A.M.R.: Writing - review & editing, Validation, Visualization. Costa, S.C.V.: Writing - review & editing, Methodology, Validation, Visualization. Costa, R.S: Writing - review & editing, Validation, Visualization. Senna, K.M.S.: Formal analysis, Conceptualization, Writing - review & editing, Supervision. Zimmermann, I.R.: Project administration, Formal analysis, Conceptualization, Data curation, Writing - review & editing, Methodology, Software, Supervision.

**It should read:**

AUTHORS’ CONTRIBUTIONS: Gonçalves, D.S.: Conceptualization, Data curation, Writing - original draft, Methodology, Software. Migowski A.: Writing - review & editing, Validation, Visualization. Costa, S.C.V.: Writing - review & editing, Methodology, Validation, Visualization. Costa, R.S: Writing - review & editing, Validation, Visualization. Senna, K.M.S.: Formal analysis, Conceptualization, Writing - review & editing, Supervision. Zimmermann, I.R.: Project administration, Formal analysis, Conceptualization, Data curation, Writing - review & editing, Methodology, Software, Supervision.

**ABSTRACT**

**Where it reads:**

It is essential to avoid advanced staging and perform surgical treatment, with emphasis on radical mastectomy.

**It should read:**

It is essential to avoid advanced stage and perform surgical treatment, with emphasis on radical mastectomy.

**METHODS**

**Where it reads:**

Cox-Shell residual analysis was conducted to assess the adequacy of the adjusted model (Supplementary Material - S2).

**It should read:**

Cox- Snell residual analysis was conducted to assess the adequacy of the adjusted model (Supplementary Material - S2).

**DISCUSSION**

**Where it reads:**

These findings are in line with previous studies that highlight the influence of staging on breast cancer survival, including metastatic ones^23-25^.

**It should read:**

These findings are in line with previous studies that highlight the influence of stage on breast cancer survival, including metastatic ones^23-25^.

**Figure 1. (A)**

**Where it reads:**

With cerebral goal

**It should read:**

With brain metastases

**Where it reads:**

No cerebral goal

**It should read:**

No brain metastases

**Figure 1. (A)**

**Where it reads:**

No goal:

With goal:

**It should read:**

No metastases

With metastases

**Table 2**

**Where it reads:**

Staging

**It should read:**

Stage

