# Supplementary material for: ERRATUM
Source: Rev Bras Epidemiol. 2023 Dec 4;26:e20230045erratum. doi: 10.1590/1980-549720230045erratum (PMC10695406; doi:10.1590/1980-549720230045erratum)
Supplement: Supplementary file 1 [file 1980-5497-rbepid-26-e20230045erratum-s1.pdf]

Supplementary Material - S1: Correlation matrix (Spearman test)

		Age at diagno-sis	Stage	BMI	Metasta-sis site	Type of surgery	Histologi-cal grade	Skin color	Hormone receptor	Trastuzu-mab exposure
Age at diagnosis	Spearman's Rho	—								
	p-value	—								
Stage	Spearman's Rho	0.053	—							
	p-value	0.542	—							
BMI	Spearman's Rho	0.064	0.019	—						
	p-value	0.463	0.825	—						
Metastasis site	Spearman's Rho	-0.090	0.001	-0.048	—					
	p-value	0.296	0.993	0.582	—					
Surgery_T	Spearman's Rho	-0.057	-0.167	0.008	0.008	—				
	p-value	0.510	0.052	0.922	0.930	—				

Footnote: * $p < 0.05$; ** $p < 0.01$; *** $p < 0.001$

Material Suplementar - S1: (continuation)

		Age at diagnosis	Stage	BMI	Metasta-sis site	Type of surgery	Histolo-gical grade	Skin color	Hormone receptor	Trastuzu-mab exposure
Histologi-cal grade	Rho de Spearman	0.125	-0.161	-0.101	0.110	0.095	—			
	p-value	0.147	0.061	0.240	0.203	0.269	—			
Skin color	Rho de Spearman	0.016	-0.073	-0.090	0.047	0.105	0.108	—		
	p-value	0.851	0.397	0.295	0.590	0.222	0.210	—		
Hormone receptor	Rho de Spearman	0.073	-0.043	-0.029	0.190 *	0.042	0.056	0.032	—	
	p-value	0.400	0.615	0.735	0.027	0.623	0.518	0.713	—	
Exp._ Trast.	Rho de Spearman	-0.070	0.375 ***	0.129	0.054	-0.029	-0.132	-0.127	0.095	-
	p-value	0.419	< 0.001	0.134	0.529	0.741	0.126	0.140	0.273	-

Footnote: * $p < 0.05$; ** $p < 0.01$; *** $p < 0.001$

Legend: Exp._Trast.: prior exposure to trastuzumab.

Supplementary Material - S2: Cox-Snell residual analysis

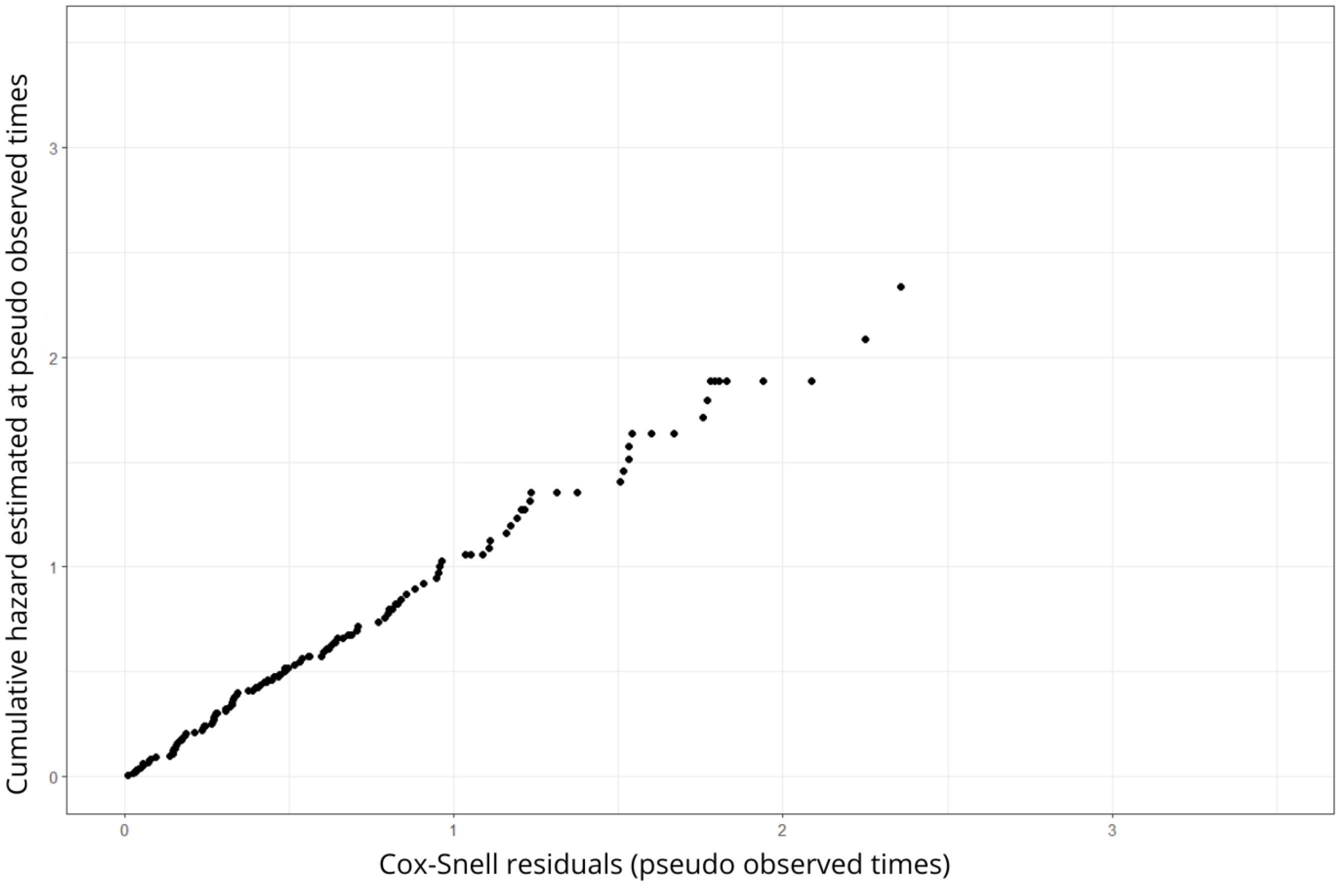

Supplementary Material - S3: Forest plot of multivariate analysis for overall survival of patients with metastatic breast cancer using trastuzumab from 09/2017 to 08/2018 in an oncology reference hospital in Rio de Janeiro, Brazil - (scenario 01)

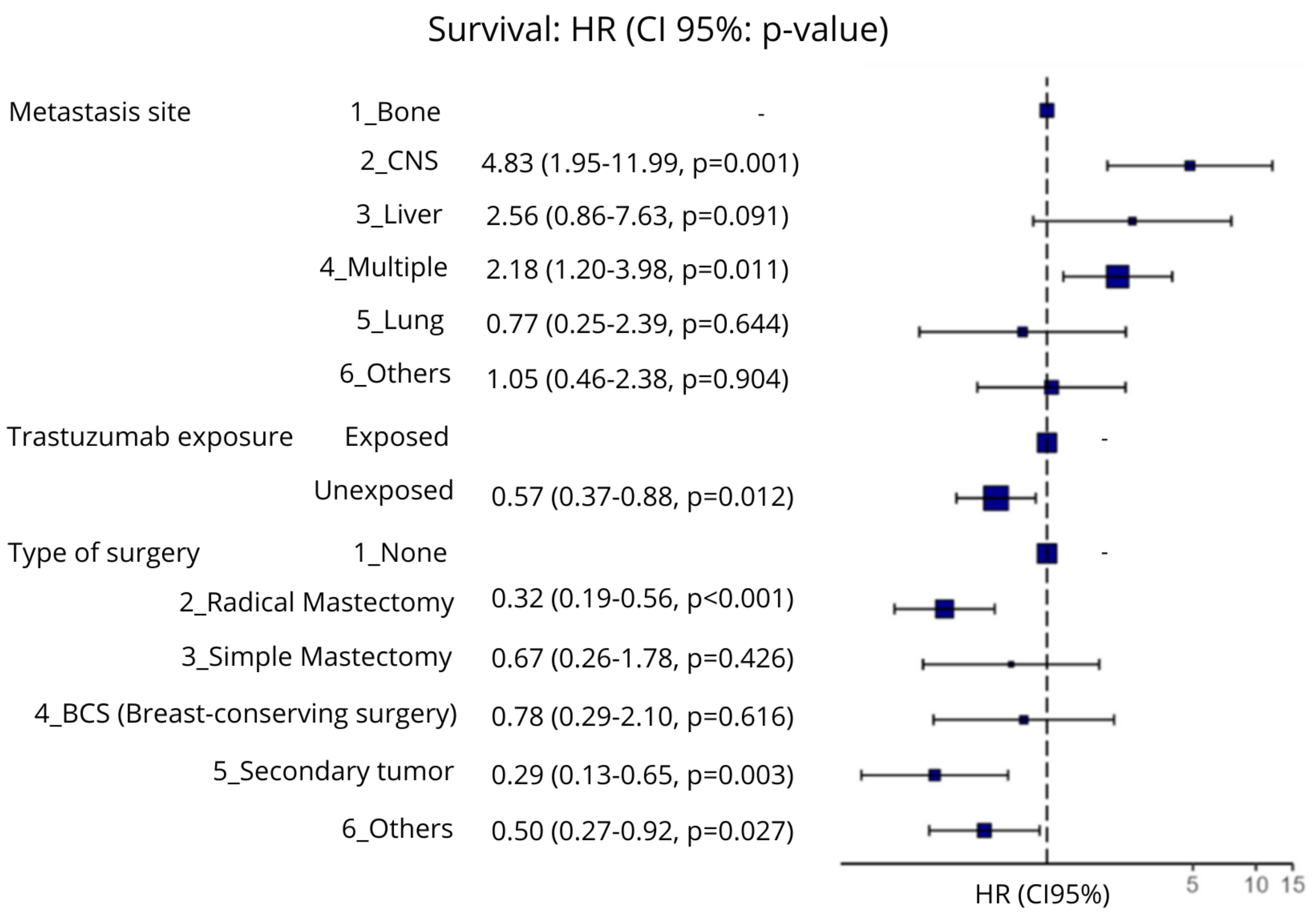

Supplementary Material - S4: Comparison between the cost of 1 mg of trastuzumab for the presentation of 440 mg in the state of Rio de Janeiro in relation to the presentation of 150 mg for the DLOG in the period from 2012 to 2021, according to the Integrated Administration System of General Services - SIASG (federal purchases) in Brazil.

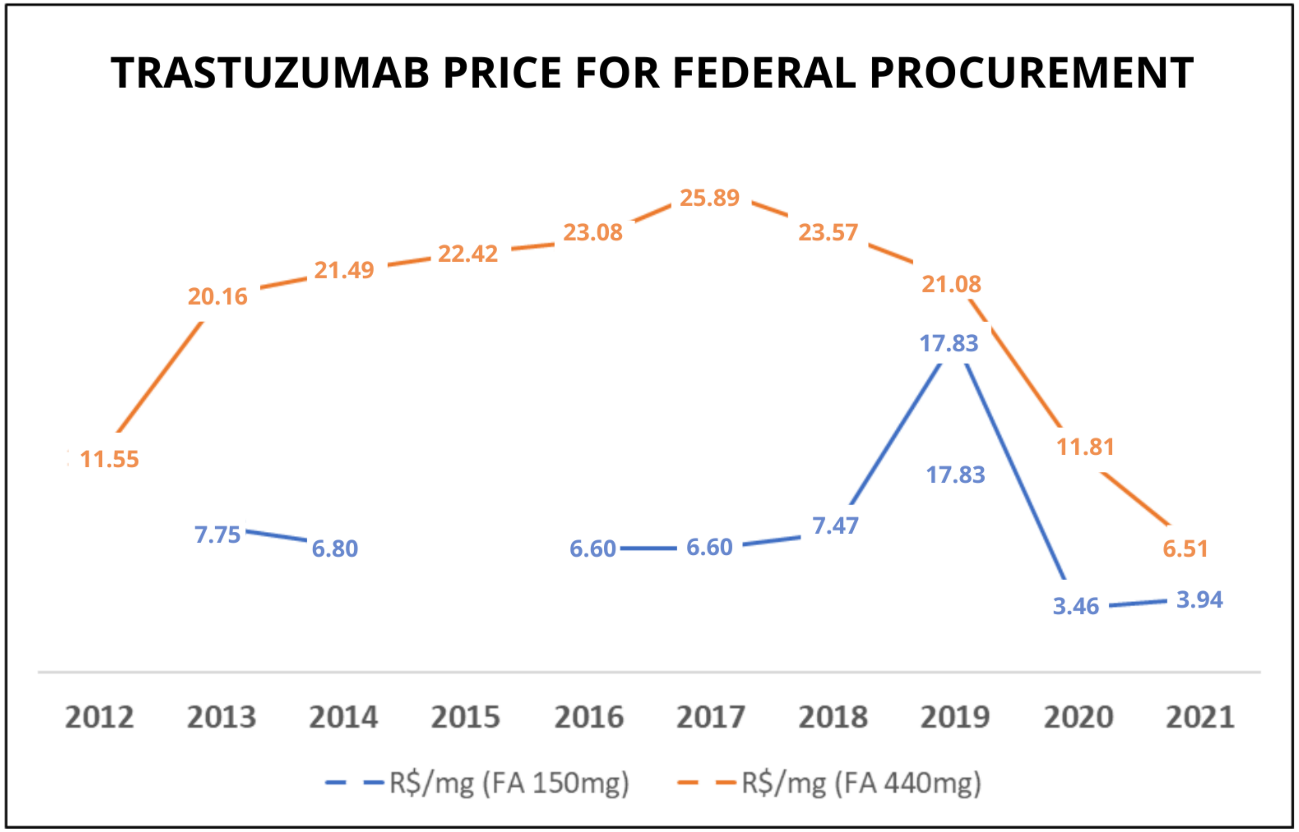

Source: Health Price Bank (BPS) - (http://bps.saude.gov.br/visao/consultaPublica/relatorios/geral/index.jsf)
